# What do cancer patients discuss online regarding CINV management? A social media-based topic modeling study

**DOI:** 10.3389/fonc.2026.1839644

**Published:** 2026-06-04

**Authors:** Hongzhan Jiang, Wanting Shen, Meng Zhou, Yinyin Lyu, Meiqi Meng, Dan Yang, Xuejing Li, Bohan Zhang, Yufang Hao

**Affiliations:** 1 School of Nursing, Beijing University of Chinese Medicine, Beijing, China; 2 Department of Nursing, Beijing Tsinghua Changgung Hospital, School of Clinical Medicine, Tsinghua Medicine, Tsinghua University, Beijing, China

**Keywords:** chemotherapy-induced nausea and vomiting, CINV, Latent Dirichlet Allocation, social media, symptom management, topic modeling

## Abstract

**Background:**

Chemotherapy-induced nausea and vomiting (CINV) remains a distressing adverse effect that compromises patients’ quality of life and treatment adherence. Traditional assessment methods often fail to capture the full scope of patients’ real-world experiences. Social media provides unfiltered patient-generated insights into symptom management and unmet needs. This study aimed to identify key themes in CINV-related discussions among cancer patients on Chinese social media platforms using natural language processing.

**Methods:**

A total of 8, 227 public posts and comments related to CINV were collected from three major Chinese social media platforms (Weibo, REDnote, Zhihu). The dataset included posts published from January 2020 to December 2025. Latent Dirichlet Allocation (LDA) topic modeling was employed to identify latent topics, followed by qualitative thematic analysis for in-depth interpretation.

**Results:**

Four themes were identified: (1) Emotional Support and Positive Mindset (30.37%), emphasizing encouragement and psychological reinforcement; (2) Symptom Management and Solution Exploration (24.05%), reflecting active pursuit of dietary and behavioral strategies; (3) Drug Knowledge and Experience Sharing (20.81%), highlighting information needs about antiemetic medications; and (4) Acute Symptom Impact and Medical Help-Seeking (24.77%), capturing severe symptom distress and use of social media to seek timely medical guidance.

**Conclusion:**

Patients’ online discussions reveal multidimensional CINV management needs beyond pharmacotherapy, including emotional support, practical coping strategies, and medication education. Social media functions as both a peer support platform and an informal resource for symptom monitoring and medical decision-making, highlighting gaps in current clinical care. These findings support the development of patient-centered, digitally informed interventions to optimize CINV management.

## 1 Introduction

Cancer remains one of the leading causes of morbidity and mortality worldwide, with an estimated 19.3 million new cases and nearly 10 million cancer-related deaths reported globally in 2020 (1). Chemotherapy continues to serve as a cornerstone of systemic anticancer treatment across a broad spectrum of malignancies (2). However, despite its therapeutic benefits, chemotherapy is associated with a constellation of debilitating side effects that substantially compromise patients’ quality of life (QoL) and treatment adherence (3). Among these, CINV is consistently ranked by patients as one of the most distressing and feared adverse effects (4, 5).

CINV can be broadly categorized into five subtypes based on the temporal pattern of onset following chemotherapy administration: acute, delayed, breakthrough, refractory, and anticipatory. Despite significant pharmacological advances in antiemetic prophylaxis, including the use of serotonin-3 (5-HT_3_) receptor antagonists, neurokinin-1 (NK-1) receptor antagonists, and olanzapine, nausea and vomiting remain inadequately controlled in a substantial proportion of patients (6). Epidemiological studies indicate that up to 40%–70% of patients subjected to moderately or highly emetogenic chemotherapy still experience varying degrees of CINV, particularly during the delayed phase (7, 8). Poorly controlled CINV can lead to nutritional deficiencies, dehydration, electrolyte imbalances, deterioration in functional status, and, in severe cases, premature discontinuation of potentially curative treatment plans (9, 10). Furthermore, emerging evidence suggests that clinicians often underestimate the incidence and severity of CINV, highlighting a significant discrepancy between healthcare providers’ perceptions and patients’ actual experiences (11, 12).

Traditional methods for assessing the symptom burden and patient needs associated with CINV, such as clinician-administered scales, standardized questionnaires, and patient-reported outcomes (PROs) derived from clinical trials, have been indispensable in deepening our understanding of this symptom. However, these conventional approaches possess inherent limitations, including recall bias and the artificial constraints of structured instruments (13). Furthermore, such tools typically collect symptom data at discrete, predetermined time points, potentially missing the dynamic, multidimensional, and context-dependent nature of symptoms as experienced by patients in their daily lives (14).

## 2 Literature review

In recent years, social media platforms have emerged as a rich, natural, and largely untapped source of patient-generated health data (15). Platforms such as online health communities and discussion forums provide spaces where patients voluntarily share real-time experiences, concerns, coping strategies, and unmet needs in their own words, free from the structural constraints of clinical measurement tools (16, 17). The analysis of such user-generated content offers several distinct advantages: it enables access to large-scale, longitudinal, and ecologically valid data; it captures the patient voice in an unfiltered manner; and it can untangle emerging themes and needs that may not have been anticipated beforehand by researchers or clinicians (18, 19). A growing body of literature has demonstrated the practical value of social media data mining in pharmacovigilance, adverse drug event detection, and the identification of patient-reported symptoms and treatment experiences across various disease contexts, including oncology (20, 21).

To systematically extract meaningful patterns from the vast and unstructured corpus of social media text data, advanced computational methods from the field of natural language processing (NLP) are essential. Among these, topic modeling has gained increasing recognition as a powerful unsupervised machine learning technique for discovering latent thematic structures within large text corpora (22). Latent Dirichlet Allocation (LDA), introduced by Blei et al. (23), is the most widely adopted generative probabilistic model for topic discovery. By modeling each document as a mixture of latent topics and each topic as a probability distribution over words, LDA can discover coherent thematic structures that capture the diversity and nuance of patient experiences without imposing predefined categories (23). This methodological advantage makes LDA a suitable tool for the present study, which aims to uncover the breadth of CINV-related concerns expressed spontaneously by patients.

In the health and biomedical domain, LDA-based topic modeling has been successfully applied to analyze electronic health records, biomedical literature, and patient narratives on social media to uncover disease-related themes, treatment concerns, and psychosocial needs (24). For instance, studies have employed topic modeling to explore patient discussions related to diabetes self-management (25), and COVID-19 vaccine hesitancy (26), yielding clinically meaningful insights that complemented traditional survey-based approaches. Within oncology specifically, recent work has demonstrated the growing utility of these methods for analyzing user-generated content on Chinese social media platforms. Bao and Chen (27) applied topic modeling to 63, 775 posts from Rednote and identified four core themes in Chinese family caregivers’ digitally expressed experience of pre-loss grief, including caregiving burden, adaptive family communication, and narratives of resilience. Similarly, Kang et al. (28) used LDA topic modeling to analyze open−ended survey responses from childhood and adolescent cancer survivors and their parents, identifying “exercise, ” “healthy diet, ” and “regular lifestyle” as common unmet needs in survivorship care. These studies collectively demonstrate that topic modeling can effectively extract clinically meaningful themes from both spontaneous social media posts and structured survey responses within oncology populations, and that such themes can inform the design of tailored supportive care and digital health interventions.

Despite the increasing application of natural language processing and social media analysis in health research, significant gaps remain. Peng et al. (29) compared outputs from social media and generative artificial intelligence, revealing that social media provides more in-depth, experience-based content. To date, few studies have leveraged patient-generated social media data to comprehensively explore the full spectrum of symptom management needs, self-care strategies, and information-seeking behaviors among cancer patients coping with CINV in real-world settings. The unique challenges posed by CINV during active chemotherapy are fundamentally distinct from the long-term lifestyle issues faced by post-treatment survivors or the grief processes experienced by caregivers. Furthermore, the spontaneous, unprompted nature of social media discourse may untangle needs not captured by structured questionnaires. The significance of this gap is threefold: (a) theoretically, CINV represents a multidimensional symptom experience that structured instruments may not fully capture, and analyzing unfiltered patient discourse can reveal novel dimensions; (b) methodologically, applying LDA topic modeling to CINV-related social media data within the Chinese healthcare context offers an opportunity to test the utility of these techniques in a new cultural and linguistic environment; and (c) operationally, understanding patients’ self-expressed needs can facilitate the design of patient-centered supportive interventions, thereby addressing current gaps in clinical practice. To bridge these gaps, this study aimed to systematically collect and analyze CINV-related posts from Chinese social media platforms using LDA-based topic modeling.

## 3 Methods

### 3.1 Study design

This study employed an explanatory sequential mixed-methods design, utilizing computational text mining and unsupervised machine learning to analyze publicly available social media data.

### 3.2 Data source

To construct a comprehensive research dataset, this study selected three representative Chinese social media platforms. These platforms have distinct focuses in terms of user demographics and content style, enabling the capture of discussions on CINV symptom management from multiple dimensions:

REDnote (https://www.xiaohongshu.com): Primarily focused on lifestyle sharing, its user base is predominantly young. It contains a large number of image-text notes regarding dietary adjustments and antiemetic experiences during chemotherapy.

Zhihu (https://www.zhihu.com): A knowledge-based Q&A platform hosting numerous in-depth questions, answers, and detailed experience-sharing articles concerning chemotherapy side effects, fostering discussions of greater depth.

Weibo (https://weibo.com): A public social media platform that reflects broad public sentiment, facilitating the capture of real-time symptom experiences and emotional expression.

### 3.3 Data collection and screening criteria

A systematic web-based data collection strategy was employed. Data were retrieved using a combination of platform-specific Application Programming Interfaces (APIs) and web scraping techniques. The temporal span of the collected posts ranged from January 1, 2020 to December 31, 2025, to capture a sufficiently large and contemporary corpus of patient-generated content, to capture a sufficiently large and contemporary corpus of patient-generated content. Data collection was conducted in January 2026. A predefined set of search queries was used to retrieve CINV-related posts, including but not limited to the following keywords and their combinations: “chemotherapy AND nausea, ” “chemotherapy AND vomiting, ” “CINV, ” “anti-nausea AND chemotherapy, ” “antiemetic AND chemotherapy.” The full list of Chinese search terms is provided as **Supplementary materials**, **Supplementary Table 1**. After applying inclusion and exclusion criteria, the final dataset comprised 8, 227 posts, with a total corpus size of approximately 1.2 million Chinese characters (mean post length: 146 characters).

Inclusion Criteria: (1) The text was written in Chinese; (2) The publisher explicitly self-identified as a cancer patient, or the content was a first-person narrative by the patient about their chemotherapy experience; (3) The content explicitly mentioned nausea, vomiting, coping strategies, or related psychological experiences during chemotherapy.

Exclusion Criteria: (1) Duplicate posts; (2) Posts that were purely advertisements, clinical trial recruitment notices, or news reposts; (3) Content that was overly brief (fewer than 20 characters) and lacked substantive information.

### 3.4 Data analysis

**Figure 1:**
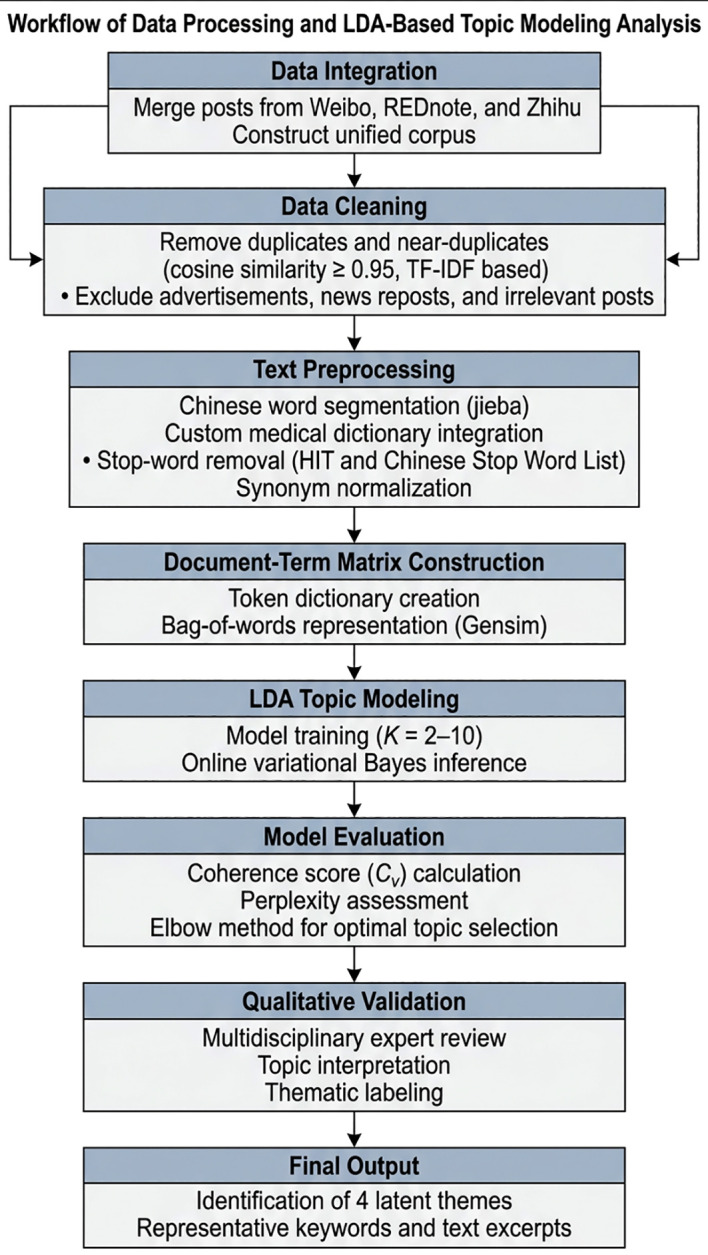
Analytical workflow.

The analytical workflow comprised four sequential stages: (a) data preprocessing; (b) LDA topic modeling; (c) determination of the optimal number of topics; and (4) qualitative thematic analysis. The complete analytical workflow is illustrated in **Figure 1**.

#### 3.4.1 Data preprocessing

All collected posts were integrated into a unified corpus and underwent a systematic data cleaning and preprocessing pipeline prior to topic modeling analysis. The preprocessing workflow was implemented in Python (version 3.10). First, duplicate and near-duplicate posts were identified and removed using a cosine similarity threshold (≥0.95) calculated based on the TF-IDF (Term Frequency-Inverse Document Frequency) vector representation of each document. Subsequently, the Chinese text data were segmented using the jieba word segmentation library in Python. To address the limitations of the jieba package in segmenting medical terminology, a custom dictionary was constructed for machine learning use. This dictionary was developed based on professional literature such as Comprehensive Medical Vocabulary and the Chinese Guideline for the Prevention and Treatment of Antineoplastic Therapy-Related Nausea and Vomiting (2023 Edition), combined with consultation feedback from clinical oncology nurses. It included chemotherapy drug names (e.g., “cisplatin”, “cyclophosphamide”), antiemetic drug names (e.g., “ondansetron”, “aprepitant”), and symptom description terms (e.g., “nausea”, “retching”, “projectile vomiting”). Finally, stop word removal was performed by referencing the Chinese Stop Word List and the Harbin Institute of Technology Stop Word List, eliminating a large number of stop words such as modal particles, conjunctions, numbers, English letters, and punctuation marks from the segmented results. Furthermore, considering the informality of user-generated natural language, common terms were subjected to synonym synthesis. Following preprocessing, a document-term matrix and the corresponding unique token dictionary were constructed using the Gensim library (version 4.3) to serve as input for the LDA model.

#### 3.4.2 LDA topic modeling

LDA was employed as the primary topic modeling algorithm. LDA is a generative probabilistic model that represents each document as a mixture of latent topics, and each topic as a probability distribution over words in the vocabulary (23). The model infers the hidden topical structure of a corpus by estimating two sets of probability distributions: (a) the document-topic distribution (θ), which describes the proportion of each topic within each document; and (b) the topic-word distribution (φ), which describes the probability of each word belonging to each topic (22, 23). The LDA model was implemented using the Gensim Python library (version 4.3) with online variational Bayes inference.

#### 3.4.3 Determination of the optimal number of topics

Selecting the optimal number of topics (K) is a critical step in LDA modeling, as an inappropriate K value may result in overly broad, redundant, or incoherent topics (30). In this study, a systematic approach combining quantitative evaluation metrics and qualitative human judgment was adopted.

First, LDA models were trained iteratively with K values ranging from 2 to 10 (in increments of 1). For each model, the following quantitative metrics were computed:

1. Coherence score (C_v): The C_v coherence measure, which evaluates the semantic similarity among the top words within each topic using a sliding window and normalized pointwise mutual information (NPMI), was calculated for each model. Higher coherence scores indicate more interpretable and semantically coherent topics. The coherence score was computed using the Gensim CoherenceModel module.
2. Perplexity: Log perplexity, a measure of how well the model predicts a held-out test set, was also computed (23). Lower perplexity values generally indicate better model generalization, although perplexity alone has been shown to correlate poorly with human interpretability (30).

The coherence scores and perplexity values were plotted against the number of topics (K) to identify candidate optimal values. The “elbow method” was applied to the coherence curve to identify the point of diminishing returns, where additional topics no longer substantially improved coherence.

Second, candidate models with the highest coherence scores were subjected to qualitative evaluation by a multidisciplinary research team consisting of two oncology nursing researchers, one computational linguist, and one oncologist. Each team member independently reviewed the top 20 keywords and representative documents for each topic in the candidate models and assessed the topics for: (a) semantic coherence and interpretability; (b) distinctiveness (minimal overlap between topics); and (c) clinical relevance and meaningfulness. It should be noted that while certain keywords may appear across multiple topics, the researchers assessed topic distinctiveness by examining representative full-text documents with high probability loadings on each topic rather than relying solely on isolated word frequency. This approach ensured that topics were evaluated based on their contextual meaning rather than superficial keyword overlap. The final number of topics was determined by consensus.

#### 3.4.4 Qualitative analysis

After the optimal LDA model was determined, each identified topic was interpreted through a structured qualitative process and assigned a descriptive label. Two researchers independently reviewed the top 20 keywords for each topic. Using the document-topic probability distribution, representative texts were extracted and analyzed to define and name the topics inductively. Subsequently, the researchers convened to compare their findings, resolving discrepancies through discussion to reach a consensus, with a third researcher arbitrating when necessary.

### 3.5 Ethical considerations

This study adhered to relevant ethical guidelines, including the Declaration of Helsinki and the guidelines of the Association of Internet Researchers (31). As the research involved a non-interventional analysis of publicly available social media data, it was exempt from formal institutional review board approval. The following measures were implemented to protect user privacy: (a) During the data preprocessing stage, all usernames, profile information, and personally identifiable information were removed; (c) No attempts were made to re-identify or contact any individual users; (b) If direct quotations from posts were included in the research findings, they were paraphrased or minimally modified to prevent source tracing via search engines while preserving the original meaning; (d) The data were stored on encrypted, password-protected local servers accessible only to authorized members of the research team.

## 4 Results

### 4.1 General characteristics

**Figure 2:**
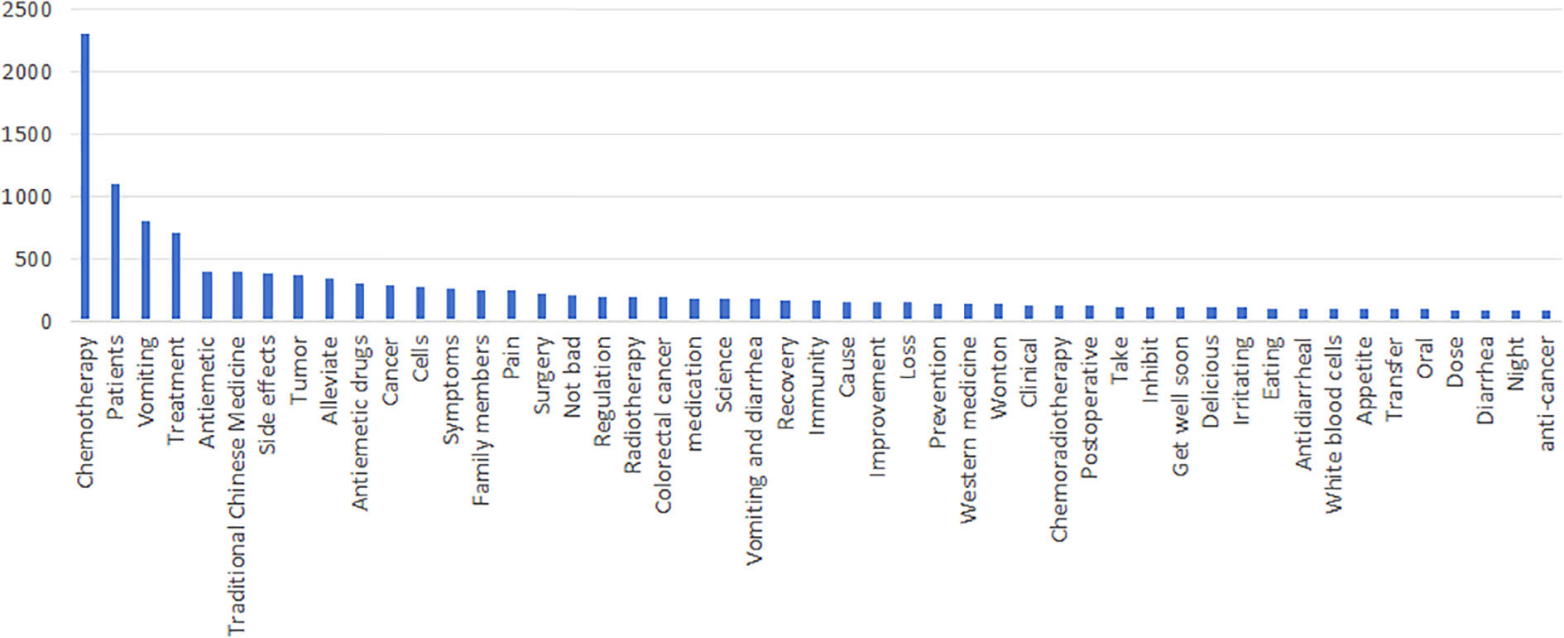
Word frequency graph-top 50.

**Figure 3:**
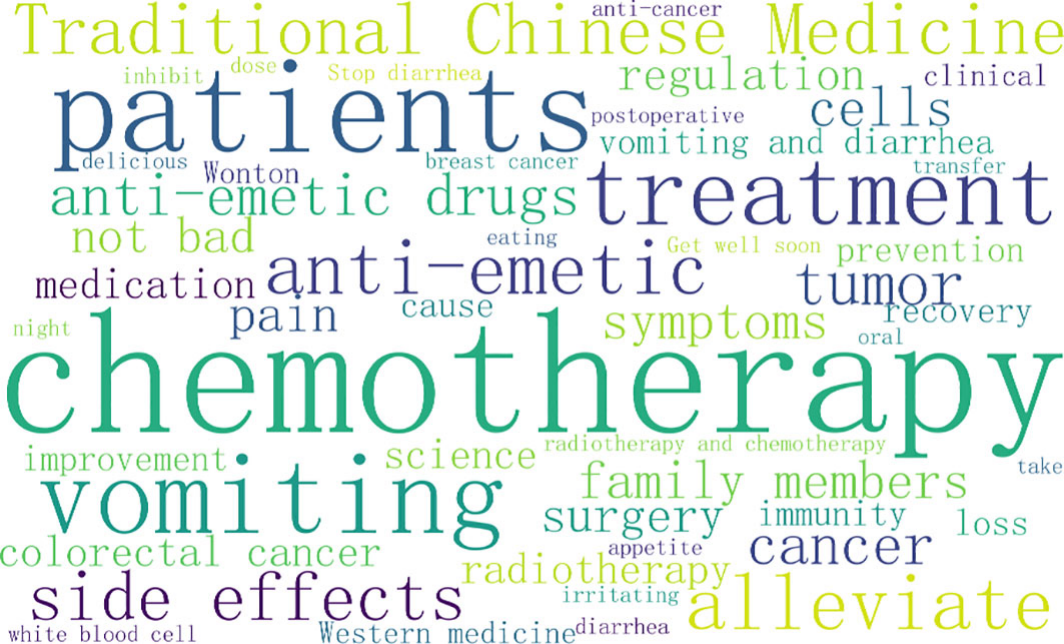
Word cloud-top 5.0.

After screening, a total of 8, 227 records of chemotherapy-induced nausea and vomiting-related questions and answers were included in this study, comprising 167 (2%) from Zhihu, 896 (10.9%) from REDnote, and 7, 164 (87.1%) from Weibo. Following word segmentation, a high-frequency word frequency analysis was conducted (**Figure 2**). The top ten most frequent words were: chemotherapy, patient, vomiting, treatment, antiemesis, traditional Chinese medicine, side effects, tumor, relief, and antiemetic drugs. Using Weiciyun, a word cloud was generated based on the top 50 high-frequency words (**Figure 3**). As illustrated in **Figure 3**, terms such as chemotherapy, vomiting, antiemesis, side effects, traditional Chinese medicine, and relief represent the primary concerns of patients, while terms such as conditioning, rehabilitation, eating, and prevention represent secondary areas of focus.

### 4.2 Results of latent theme analysis

The LDA topic model identified four topics, with each topic and its corresponding top 20 keywords presented in **Table 1**. This optimal number of topics was determined through a comprehensive integration of quantitative and qualitative data. Initially, the coherence scores (**Supplementary materials**, **Supplementary Figure 1**) and perplexity scores (**Supplementary materials**, **Supplementary Figure 2**) for models with 2 to 10 topics were calculated. Higher coherence scores indicate better semantic quality. Although the model with three topics exhibited a higher coherence score, it was considered insufficiently granular based on qualitative assessment. Following team discussion, the model with four topics was selected as it better aligned with the objectives of the present study.

**Table 1:** Summary of all 4 latent themes, with keywords and prevalence.

Theme	Keywords	Prevalence (n=7829), n(%)
Theme 1: Emotional Support and Positive Mindset	Come on, feeling, attention, good, state, blogger, try, cute, happy, delicious, wonton, effective, improvement, great, joyful, snicker, tasty, looks like, recover, victory	2378 (30.37%)
Theme2:Symptom management and solution exploration	Chemotherapy, remission, diet, plan, effect, surgery, metastasis, gastrointestinal tract, discomfort, eating, health, recovery, method, rectal cancer, sadness, joy, pathology, cooking, lymph, sunshine	1883 (24.05%)
Theme 3: Drug Knowledge and Experience Sharing	Drugs, nutrition, side effects, share, rehabilitation, appetite, toxicity, flowers, light (in taste), like, vitamins, persist, nerves, consult, be careful, disperse, optimistic, TV dramas, sun, take care of	1629 (20.81%)
Theme 4: Acute Symptom Impact and Medical Help-Seeking	vomiting, food, cells, cancer, body, hospital, radiotherapy, hug, severe, help, keep, hope, Western medicine, life, comfortable, rest, recurrence, course of treatment, diagnosis, drinking water	1939 (24.77%)

### 4.3 Qualitative analysis of the 4 themes

We conducted a qualitative interpretation of each of the four themes, supplemented with representative quotations.

#### 4.3.1 Theme 1: Emotional support and positive mindset (30.37%)

This theme is characterized by a strong prevalence of positive emotional expression. Positive emotion words such as “jia you” (meaning “stay strong” or “keep going”) have emerged as the most representative keywords, indicating that patients experiencing CINV have a strong need for emotional encouragement and psychological support.

“Whenever I feel down, I remind myself that this is only temporary, and a bright future is waiting for me. Keep going!”

“Watching you document your life so positively has really encouraged my mother a lot. Let’s keep going together, for the ones we love!”

#### 4.3.2 Theme 2:Symptom management and solution exploration (24.05%)

This theme centers on the symptoms experienced during chemotherapy, with the keywords exhibiting a clear problem-solving orientation. Terms such as “relief, ” “solution, ” “effect, ” and “method” indicate that patients are actively seeking specific strategies to manage CINV, particularly information related to daily dietary management.

“How to prevent nausea and vomiting after returning home from chemotherapy.”

“What do you all eat after chemotherapy? My dad feels nauseous and has no appetite. I want to make him something light yet nutritious. Please recommend some!”

#### 4.3.3 Theme 3: Drug knowledge and experience sharing (20.81%)

“Excuse me, everyone. The doctor told me to take dexamethasone before chemotherapy. Should I take it on an empty stomach or after meals? Taking it before meals makes my stomach feel uncomfortable.”

“When is the best time to take Aprepitant for better effect? I’d like to follow your example too.”

“I heard dexamethasone is also part of the antiemetic regimen, but I’m worried about the side effects of hormones. I’m really torn.”

#### 4.3.4 Theme 4: Acute symptom impact and medical help-seeking (24.77%)

This theme focuses on the intense distress experienced by patients during the acute phase of symptoms, as well as their pursuit of professional medical support when symptoms become severe.

“Last year, I suffered from extremely severe vomiting after chemotherapy. I felt as if my stomach was coming up, and it was absolutely unbearable. I went to the emergency room in the middle of the night, and only felt better after the doctor gave me an antiemetic injection.”

“These past few days, I’ve had severe chemotherapy side effects. I throw up whatever I eat or drink, even water, and I feel like I’m about to collapse. I went to the hospital for IV nutrition yesterday and feel a little better today.”

## 5 Discussion

### 5.1 The diversity of CINV symptom management needs in cancer patients undergoing chemotherapy

Based on LDA topic modeling analysis of discussions related to CINV among cancer patients on social media platforms, this study identified four core themes. These are: emotional support and positive mindset (Theme 1), symptom coping and regimen discussion (Theme 2), medication knowledge and experience sharing (Theme 3), and acute symptom distress and medical assistance seeking (Theme 4).

According to Wilson’s theory of information behavior, information needs arise in response to problems within a specific context and are closely related to physiological, cognitive, and affective needs (32). The findings of this study are highly consistent with this theory: Theme 2 and Theme 4 primarily reflect patients’ physiological needs during the acute phase of CINV; Theme 3 reflects patients’ cognitive needs for medication knowledge and refined management; Theme 1 focuses on presenting patients’ emotional needs. This suggests that healthcare professionals should address multidimensional care needs when managing CINV symptoms. Specifically, a tiered CINV care model could be implemented: during the acute phase, a standardized risk-screening tool and rapid-response antiemetic protocol should be in place; during the inter-treatment period, structured educational materials on dietary management, medication adherence, and coping strategies should be provided through digital platforms or nurse-led follow-ups; and across all phases, brief psychological screening and referral pathways for emotional support services should be integrated into routine oncology care. Such a model would bridge the identified gaps between patients’ expressed needs and current clinical practice.

### 5.2 Emotional support as a profound psychosocial need in CINV management

This study found that emotional support and a positive mindset are frequently expressed by patients on social media platforms. Patients not only describe symptoms such as nausea and vomiting but also commonly use these platforms to seek encouragement, comfort, and companionship. Previous studies have indicated that nausea and vomiting are among the most feared adverse effects for chemotherapy patients, significantly impacting their quality of life, treatment experience, and adherence to therapy (33). It should be noted, however, that recent social media-based research has found that alopecia may be the most frequently searched chemotherapy-related side effect (34). This suggests that while hair loss garners greater search volume—possibly due to its visible and socially impactful nature—CINV remains a profoundly distressing experience that drives patients toward seeking emotional support and coping strategies through sustained online discussion, as captured in our dataset. In particular, nausea, with its strong subjective experience and prolonged duration, is often more difficult to control than vomiting and more likely to trigger emotional distress (35). The emotional interactions observed on social media reflect a strong unmet need for psychological support beyond the conventional healthcare system. For cancer patients, support from peers is particularly valuable due to its experiential authenticity and situational relevance, compensating for the limitations of healthcare professional support in terms of daily-life applicability and immediacy (36).

### 5.3 Patients lack adequate strategies for managing symptoms

The theme of symptom coping and strategy discussion reveals that patients are not merely passively venting on social media platforms but are actively exchanging methods to alleviate nausea and vomiting. This indicates that patients have developed a strong awareness of proactive symptom management and seek coping strategies that are more closely aligned with their daily life scenarios, beyond standard medical advice. This finding aligns with the current clinical reality of CINV. Although international guidelines provide well-established recommendations for prophylactic antiemetic therapy based on the emetogenic risk (high, moderate, low) of chemotherapy regimens (37), a significant proportion of patients in real-world settings still experience inadequate control of nausea and vomiting in clinical practice (38). This is particularly true for delayed nausea, breakthrough nausea, and anticipatory nausea, which are more challenging to manage effectively. While pharmacological prophylaxis remains the cornerstone, dietary management, activity scheduling, and behavioral adjustments during the home-based phase are also issues of high concern to patients.

### 5.4 Patients have an urgent need for knowledge related to antiemetic medications

Drug knowledge (Theme 3) was a high-frequency keyword mentioned by patients. This indicates that patients’ engagement with CINV drug management extends beyond merely whether antiemetics were prescribed; they desire to understand the mechanisms of action, grasp the principles of use, and participate in treatment decision-making. From a clinical perspective, standardized prevention of CINV emphasizes risk stratification and combination therapy strategies, including the rational combination of drugs such as 5-HT3 receptor antagonists, NK1 receptor antagonists, dexamethasone, and olanzapine (39). However, patient discussions on social media indicate that prescribed treatment plans are not equivalent to being fully understood. Such knowledge gaps may lead to poor medication adherence. Study by Hyvert et al. (40) has demonstrated that the level of medication knowledge serves as a critical mediating variable influencing medication adherence.

Our analysis of Theme 3 revealed that patients not only sought factual information about antiemetic medications but also actively shared personal experiences with specific drugs, compared side effects, and discussed preferences regarding trade-offs between efficacy and tolerability. These observed patterns of peer-to-peer deliberation about medication choices suggest that patients desire a more active role in treatment decisions. Shared decision-making in CINV management aligns with broader recommendations for patient-centered cancer care (41); our findings suggest that such approaches should extend to antiemetic selection, particularly when balancing symptom control with quality-of-life considerations.

### 5.5 Social media serves as an important channel for patients to identify risks and seek medical advice

Theme 4 revealed that some patients immediately post on social media when their symptoms acutely worsen, describing persistent vomiting, difficulty eating, signs of dehydration. This finding suggests that patients require not only routine health education but also clear, actionable risk warning information (41). Concurrently, this theme highlights the potential for digital health support. If the high-frequency, acute help-seeking content related to CINV on social media could be transformed into structured health education resources or integrated with hospital-based internet follow-up and symptom reporting systems, it might enhance the capacity for early identification and timely intervention. However, the potential downsides of such integration must also be carefully considered. An influx of low-priority or non-urgent symptom reports could overwhelm clinical resources and obscure genuinely urgent cases. Compared to traditional PROs, social media data and PRO tools serve complementary, rather than competitive, roles. While PROs provide standardized, validated, and operationally relevant measures for clinical monitoring, social media analytics capture spontaneous, unprompted patient narratives that may reveal concerns not anticipated by structured instruments.

### 5.6 Practical implications for clinical CINV management

These findings can inform several concrete clinical strategies: (a) incorporating brief emotional screening and peer-support referrals into CINV management protocols; (b) developing structured, patient-centered educational materials on dietary and behavioral coping strategies; (c) enhancing medication counseling to address the knowledge gaps identified in online discussions, particularly regarding administration timing and side-effect expectations; and (d) exploring digital symptom-monitoring tools with clear escalation pathways for acute-phase distress.

## 6 Limitations

Several limitations should be acknowledged in this study. First, although the data were collected from China’s three major social media platforms, posts from Sina Weibo were predominant (7, 164 out of 8, 227 posts), This imbalance may bias the topic distributions toward themes more prevalent in real-time, emotionally expressive short-form content, potentially underrepresenting the more detailed narratives characteristic of REDnote and Zhihu. Consequently, the generalizability of the study findings may have been affected. Second, the user identity was inferred from self-reported content rather than verified clinical records, which may introduce potential misclassification bias. Some posts may have been authored by caregivers or family members, and their perspectives, while valuable, may differ from patients’ own experiences. Third, despite our use of a custom medical dictionary to improve the jieba segmentation tool, biases related to tokenization remain. Slang expressions, context-dependent meanings, and metaphorical language commonly used on social media may not have been fully captured or correctly segmented, potentially influencing topic generation and interpretation.

## 7 Conclusion

This study employed LDA-based topic modeling to systematically analyze patient-generated social media data on CINV, revealing four distinct thematic domains: emotional support and positive mindset, symptom management and solution exploration, drug knowledge and experience sharing, and acute symptom impact with medical help-seeking. These findings illustrate that CINV management needs extend beyond pharmacological control to encompass emotional support, practical coping strategies, and medication-related education. Social media serves not only as a platform for peer support but also as an informal resource for symptom monitoring and medical decision-making, highlighting gaps in current clinical care. By capturing the patient voice in an unfiltered, real-world context, this study provides a complementary perspective to traditional clinical assessments and underscores the importance of developing multidimensional, patient-centered interventions. Future efforts should focus on integrating patient-generated insights into supportive care frameworks, leveraging digital tools to enhance health education, symptom monitoring, and timely intervention, ultimately improving the quality of life and treatment adherence for patients undergoing chemotherapy.

## Data Availability

The original contributions presented in the study are included in the article/**Supplementary Material**. Further inquiries can be directed to the corresponding authors.
