# Supplementary material for: What do cancer patients discuss online regarding CINV management? A social media-based topic modeling study
Source: Front Oncol. 2026 Jun 4;16:1839644. doi: 10.3389/fonc.2026.1839644 (PMC13275380; doi:10.3389/fonc.2026.1839644)
Supplement: Supplementary file 1 [file DataSheet1.docx]

Supplementary materials

Table S1 Complete list of search queries used to retrieve CINV-related posts from Chinese social media platforms

| Chinese search strategy | English description |
| --- | --- |
| 化疗 AND 恶心 | chemotherapy AND nausea |
| 化疗 AND 呕吐 | chemotherapy AND vomiting |
| 化疗 AND 想吐 | chemotherapy AND “feel like vomiting” (colloquial) |
| 化疗 AND 反胃 | chemotherapy AND “upset stomach” / retching |
| 化疗 AND 恶心呕吐 | chemotherapy AND “nausea and vomiting” |
| 化疗 AND 呕吐物 | chemotherapy AND vomitus |
| CINV | CINV (English abbreviation used by patients) |
| 止吐药 AND 化疗 | antiemetic AND chemotherapy |
| 抗吐 AND 化疗 | anti-vomiting AND chemotherapy |
| 抗恶心 AND 化疗 | anti-nausea AND chemotherapy |
| 化疗 AND 食欲 | chemotherapy AND appetite |
| 昂丹司琼 AND 化疗 | ondansetron AND chemotherapy |
| 阿瑞匹坦 AND 化疗 | aprepitant AND chemotherapy |
| 地塞米松 AND 止吐 | dexamethasone AND antiemetic |


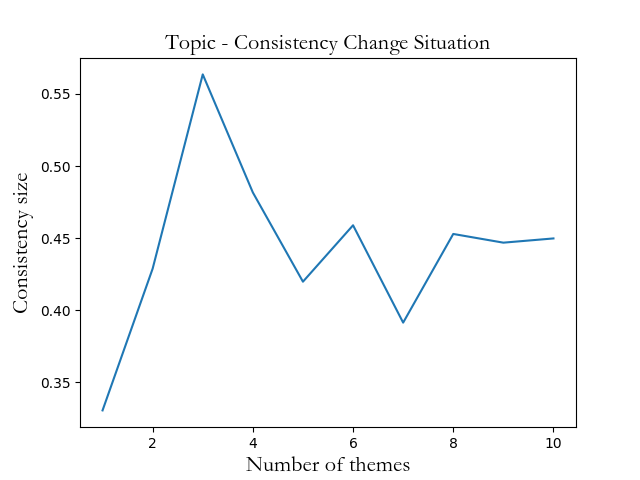


Fig S1 Coherence scores


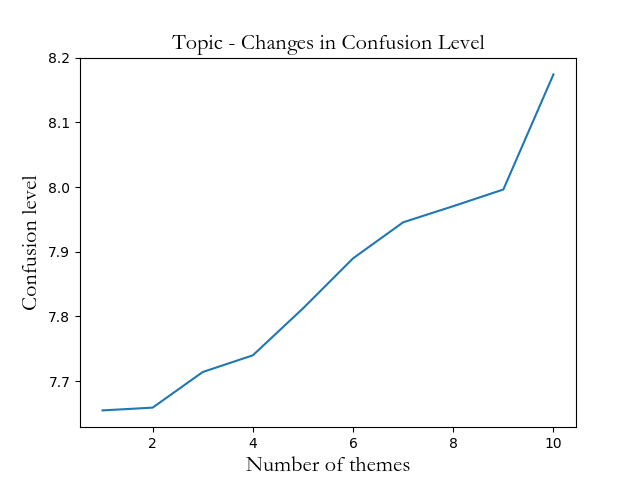


Fig S2 Perplexity scores
